# Malaria in the ‘Omics Era’

**DOI:** 10.3390/genes12060843

**Published:** 2021-05-30

**Authors:** Mirko Pegoraro, Gareth D. Weedall

**Affiliations:** School of Biological and Environmental Sciences, Liverpool John Moores University, Liverpool L3 3AF, UK; M.Pegoraro@ljmu.ac.uk

**Keywords:** *Plasmodium*, malaria, genomics, methylomics, methylation

## Abstract

Genomics has revolutionised the study of the biology of parasitic diseases. The first Eukaryotic parasite to have its genome sequenced was the malaria parasite *Plasmodium falciparum*. Since then, *Plasmodium* genomics has continued to lead the way in the study of the genome biology of parasites, both in breadth—the number of *Plasmodium* species’ genomes sequenced—and in depth—massive-scale genome re-sequencing of several key species. Here, we review some of the insights into the biology, evolution and population genetics of *Plasmodium* gained from genome sequencing, and look at potential new avenues in the future genome-scale study of its biology.

## 1. Introduction

Since the genome of the malaria parasite *Plasmodium falciparum* was published in 2002 [1], alongside that of its mosquito vector [2] and its human host (Consortium and International Human Genome Sequencing Consortium, 2001) [3], malaria genomics has led the way in the study of eukaryotic pathogens. Since then, a growing number of *Plasmodium* species’ genomes have been sequenced and large-scale population resequencing studies have been carried out in *P. falciparum* and several other species. These efforts have allowed the evolutionary and population genomics of malaria parasites to be studied in unprecedented detail and provide a model for the application of genomics to the study of other parasite species. The application of other genome-wide sequencing analyses (e.g., transcriptomics and methylomics) broadens our knowledge of *Plasmodium* biology and presents new frontiers to study. Here we consider some of these and how they can be studied at scale. This review presents some of the key advances in the study of the evolutionary genomics of malaria parasites made possible by the application of genomic sequencing technologies.

## 2. Expanding Horizons: Sequencing Across the Genus *Plasmodium*

**Figure 1:**
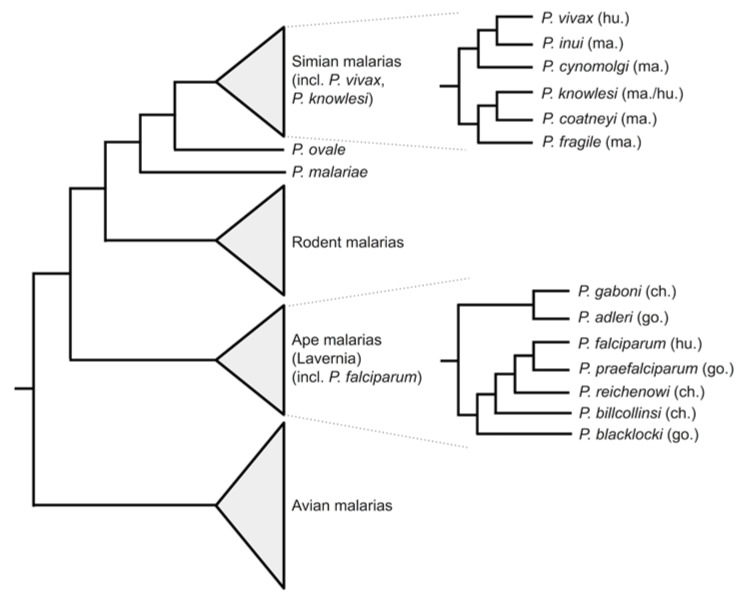
Phylogeny of malaria parasites, showing the relationships among human-infecting species. The tree is a schematic diagram indicating topology (branch lengths are not to scale). The phylogenetic positions of human-infecting *Plasmodium* species are indicated. More detailed subtrees are shown for major lineages of simian malaria parasites and for the Laverania subgenus of ape parasites. Host species are indicated in brackets (hu = human; ma = macaque; ch = chimpanzee; go = gorilla). Tree topologies are after [4], and after [10] for the Laverania.

The genus *Plasmodium* contains a huge number of species that parasitise a wide range of vertebrate hosts (Figure 1). For a broad view of the phylogenetic relationships among the malaria parasites, see [4]. At the time of writing, 5-6 *Plasmodium* species are known to infect humans. Four of these have been known for many years: *Plasmodium falciparum* and *Plasmodium vivax* are the most widespread and clinically important species, with *Plasmodium malariae* and *Plasmodium ovale* both less common and less well studied. More recently, these have been joined by *Plasmodium knowlesi*, a zoonotic malaria that primarily infects macaques, and *P. ovale* has been recognised as not one but two (probable) species: *P. ovale curtisi* and *P. ovale wallikeri* [5].

*Plasmodium* genomes are, in some ways, rather attractive genomes to sequence: haploid in the vertebrate host (and in vitro culture), not too large (around 23 Mb, encoding around 5500 genes for *P. falciparum*) and with 14 well-defined chromosomes. In other ways they present technical challenges: they are extremely AT-rich, the resulting lowered complexity of parts of the genome increasing the challenge in sequencing and assembly. However, these challenges have been overcome and as a result, a large and growing number of *Plasmodium* species’ genomes have been fully sequenced and assembled, to varying levels of completeness. For some reference genomes, initial sequencing and assembly efforts using first- and second-generation sequencing technologies have subsequently been improved using third-generation, long read sequencing technology. Additional strains have also been independently sequenced and assembled for several species [6,7,8,9].

Alongside *Plasmodium falciparum* [1], the other human malaria parasites *P. vivax* [6,11], *P. malariae*, *P. ovale curtisi* and *P. ovale wallikeri* [12,13,14] have been sequenced. Other members of the subgenus Laverania, parasites of African great apes, have been sequenced including the chimpanzee parasites *P. reichenowi* and *P. adleri* and gorilla parasites *P. gaboni*, *P. blacklocki* and *P. billcollinsi* [10,15]. A *P. vivax*-like parasite of great apes has also been sequenced [13]. The simian parasite of macaques and zoonotic human pathogen *P. knowlesi* has been sequenced [16,17], as well as other simian parasites including *P. coatneyi* [18] and *P. cynomolgi* [8,9]. Outside of the primate malarias, the genomes of rodent malaria parasites *P. berghei*, *P. chabaudi chabaudi* and *P. yoelii yoelii*, important model species for the study of malaria biology, have been sequenced [7,19,20,21,22]. Two avian malaria species, *P. gallinaceum* and *P. relictum*, have been sequenced [23]. Additional strains have also been independently sequenced and assembled for *P. falciparum* [24], *P. vivax* [6,11] and *P. cynomolgi* [8,9]. Together, this represents an enviably richly sequenced genus.

Having so many species’ genomes provides a rich resource for studying evolution in the genus. For example, an early study of comparative structural genomics between *P. falciparum* and rodent malaria parasites showed extensive chromosome structural rearrangement and that lineage-specific genes could often be found at the breakpoints of these translocations [25]. More recently, third-generation long read sequencing has provided further insights into the genomic architecture of *Plasmodium*. The central region of each chromosome, around the centromere, comprises a relatively stable ‘core genome’ characterised by broad co-linearity and one-to-one homology of the genes among genomes. An estimated 90% [26] or more [24,26] of the genome has been suggested to be ‘core genome’. The outer ‘subtelomeric regions’ are much more variable and are enriched for large and hypervariable gene families such as the variant surface antigens *var*, *rifin* and *stevor* [25,27,28,29,30], likely due to increased activity of processes like slipped-strand mispairing and ectopic recombination generating new genes and segmental duplications. The repetitive nature of the subtelomeric regions made them particularly difficult to study until long read sequencing technologies became more advanced [16,24]. The insight afforded by these advances is important because of the key roles played by some of these gene families in the infection biology of the parasite, such as immune evasion and sequestration of infected red blood cells. Large multi-gene families show different distributions among *Plasmodium* species; for example, the *kir* and *SICAvar* expanded gene families of *P. knowlesi* are not confined to the subtelomeres but occur throughout the genome [16,17]. Links between the structural genomics and the biology of different *Plasmodium* species will be an interesting area of study made possible by the advances in sequencing technologies.

A densely sequenced genus has other advantages. Comparative genomics among more closely related species allows the dating of gene family expansions [10,31] and the identification of processes such as introgression and horizontal gene transfer [10,32,33], both of which can play major roles in adaptation and virulence. Among members of the Laverania, one introgression event is of particular interest. It transferred orthologues of members of a key erythrocyte invasion complex (*rh5* and *cyrpa*) from an ancestor of the gorilla parasite *P. adleri* to the lineage leading to *P. falciparum* and its sister species, the gorilla parasite *P. praefalciparum* [10,32,33]. These genes, along with the proteins P113 and RIPR form a protein complex that is essential for red blood cell invasion [34,35,36,37]. RH5 binds the erythrocyte surface protein Basigin and displays differential binding efficiency to human, gorilla and chimpanzee Basigin, implicating it as a key determinant of host specificity [38,39,40] and a vaccine candidate [41].

Identification of cryptic or incipient species is the point where inter-specific comparative genomics meets population genomics, discussed in the next section. Two notable examples here are the human parasite *Plasmodium ovale* and the simian parasite *Plasmodium knowlesi*. *Plasmodium ovale* is now recognised to be two distinct, non-recombining lineages, called *Plasmodium ovale curtisi* and *Plasmodium ovale wallikeri* [5]. Recognition of this previously cryptic diversity is evolutionarily significant, but also relevant to the clinical manifestations of infection. For example, *Plasmodium ovale wallikeri* displays a shorter latency period (time to onset of symptoms, linked to the dormant liver hypnozoite life-cycle stages) and deeper thrombocytopaenia (low blood platelet levels) than *Plasmodium ovale curtisi* [42,43,44,45]. In addition, where malaria transmission is seasonal, as it is in much of West Africa, latency periods are significantly lower during transmission season [42,43]. *Plasmodium knowlesi*, which naturally infects macaques but can be transmitted to humans, is found across southeast Asia following the distribution of its macaque hosts. Population genomics indicates both putative cryptic lineages and large-scale introgression, discussed in the next section.

## 3. The Variable Genome: Genomic Diversity within *Plasmodium* Species

**Figure 2:**
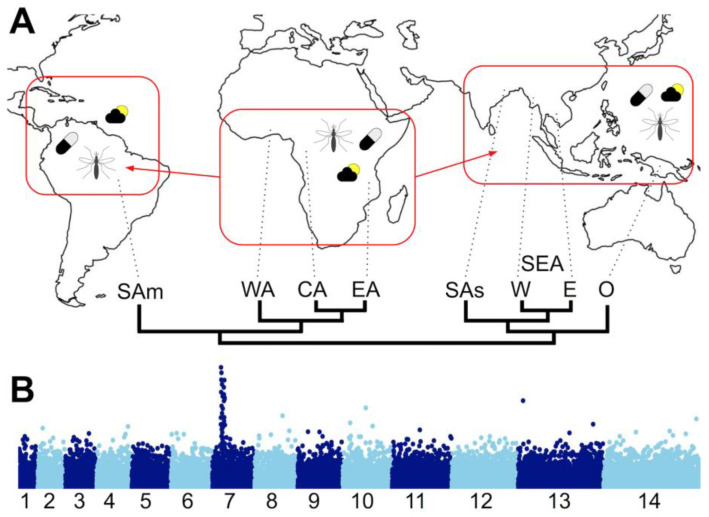
*Plasmodium falciparum* population genomics. (**A**) Map representing spread of the globally distributed species *P. falciparum* from sub-Saharan Africa to South America and Asia and the differing selection pressures that may drive local adaptation to different settings within and between continents: climatic and environmental conditions (sun and cloud); different vector species (the mosquito); differing treatment regimes (the pill). Below the map is a schematic population structure tree based on genome-wide variation data, after [48]. (Regional populations are indicated at the tips of the tree: SAm = South America; WA = West Africa; CA = Central Africa; EA = East Africa; SAs = South Asia; SEA,W = South East Asia, West; SEA,W = South East Asia, East; O = Oceania). (**B**) Representation of a genome-wide scan for signatures of selection. Indices (e.g., homozygosity or linkage disequilibrium within a population, *F_ST_* among populations, etc.) are plotted on the y-axis across all chromosomes on the x-axis (here, for *P. falciparum*’s 14 chromosomes) to identify outlier regions, such as the chloroquine resistance transporter gene (*crt*) region on chromosome 7 shown here. Data points could be individual variant sites, genes or other chromosomal regions, depending upon the index being plotted. By highlighting outliers compared to a genomic average, confounding effects of population histories can be avoided.

Population level re-sequencing of *Plasmodium* genomes has allowed population genomics to be studied in great detail, both to identify geographical population structure and genome regions under various selection pressures including anti-malarial drugs and the host’s adaptive immune response (Figure 2). For an excellent review of malaria population genomics, see [46]. The large-scale data-sharing Malaria Genomic Epidemiology Network (MalariaGEN) has made available thousands of parasite genomes—7000 *P. falciparum* genomes from 28 countries at the time of writing—to the research community [47,48]. The data provide an unprecedented snapshot of *P. falciparum* population genetics and the associated advances in sample preparation, sequencing technology, analysis methods and data sharing lay the foundations to more fully integrate genomics into disease epidemiology, though technical challenges remain [49,50].

*P. falciparum* arose in Africa. Until recently, it was thought to have evolved from an ancestor that infected the common ancestor of humans and chimpanzees, and to have speciated with its hosts (into *P. falciparum* in humans and *P. reichenowi* in chimpanzees). More recent sampling of the Laverania subgenus suggests a likely cross-species transmission from gorillas, as its closest relative is the gorilla parasite *P. praefalciparum* [51,52]. Perhaps due to this recent host switch the historical *P. falciparum* populations underwent a population bottleneck that is evident today in the low level of genetic diversity present in the species, much lower than in the other Laverania [10,52,53], or in *P. vivax* [54].

Before genomic data were available for *P. falciparum*, microsatellite genotyping showed a population structure reflecting current transmission intensities in different parts of the world [55]. For example, extensive outcrossing occurs in many parts of sub-Saharan Africa where transmission intensity is high, as the obligately sexual parasite more frequently encounters gametes from other parasites in the mosquito host. This leads to extremely low linkage disequilibrium (LD) even between genes in close proximity on a chromosome and to low divergence (as measured by *F_ST_*) among populations. In other regions where transmission rates are lower, mixed-genotype infections are rarer and selfing is more the norm leading to LD over a greater range on chromosomes and to higher *F_ST_* among populations [55]. The increased resolution of whole-genome population resequencing allowed the identification of fine population structure and the recognition of different populations [56]. For example, across sub-Saharan Africa, genome-wide genetic diversity defines East, West, Central and other ancestral parasite populations [48,56,57], including a highly divergent population in the Horn of Africa [57].

Alongside whole genome resequencing, still relatively expensive despite falling costs, the development of other ‘post-genomic’ tools such as SNP ‘barcoding’ panels [58,59,60] has allowed parasite population structures to be studied. This approach has been used in a number of studies of *Plasmodium vivax* [61,62,63,64,65,66]. *P. vivax*, like *P. falciparum*, may have originated in Africa and be of gorilla origin [52], though some lines of evidence support a simian origin in Asia [67]. Today *Plasmodium vivax* is the most geographically widespread of the human malarias, and accounts for an increasing proportion of malaria cases in many endemic areas [68]. The species is more genetically diverse than *P. falciparum* [54,69] which may partly reflect low diversity and a more recent bottleneck in *P. falciparum*, but may also partly reflect its distinctive population structure. In co-endemic regions, the population structures of *P. vivax* have often been seen to differ from *P. falciparum*, being more genetically diverse and less geographically structured [65,69,70,71]. The population genetics of *P. vivax* varies across its range. In Latin America high levels of differentiation are seen among sites, suggested to indicate multiple independent introductions of the parasite [72,73,74,75]. In southeast Asia, genetic diversity tends to be high, with lower differentiation among different regions and countries [72,73,74,75,76]. Little population structure and high genetic diversity is seen in the south Pacific [69,77,78]. *P. vivax* is rare, though not absent, across much of Africa [79] due to the prevalence of the Duffy antigen negative blood group in many African populations; the Duffy antigen being the primary receptor required for erythrocyte invasion [79,80]. African endemic foci are found in Madagascar and the Horn of Africa in the east [79,80,81] and Mauritania in the west [61,62,63], with patterns of genetic diversity and divergence from geographically distinct populations indicating long-lived, endemic populations [61,62,63]. The contrast between *P. vivax* and *P. falciparum* in co-endemic regions (e.g., [65,71]) is significant because the fracturing of parasite populations into smaller, distinct, isolated subpopulations, fewer polyclonal infections, less outcrossing, increased linkage disequilibrium and reduced genetic diversity are all indicators of falling endemicity due to successful disease control. Therefore, the absence of these indicators in *P. vivax* in some settings suggests a greater resilience of the species to malaria control interventions. In some cases *P. vivax* appears to retain, somewhat paradoxically, high genetic diversity even with high LD [82] or declining transmission [83], whether as a result of more recent populations fracturing, epidemic transmission patterns, the effects of selection or the effects of relapse from dormant hypnozoites is not fully determined.

The population structure of the simian parasite and human zoonosis *Plasmodium knowlesi* has been best studied in Bornean and Peninsular Malaysia. The parasite shows highly differentiated subgroups, broadly associated with their natural vertebrate host species: long tailed (*Macaca fascicularis*) and pig tailed (*Macaca nemestrina*) macaques [84]. Both are found in human infections in Bornean Malaysia [85,86,87,88]. A third divergent population is documented in both humans and macaques on Peninsular Malaysia [89,90,91]. Further substructure is seen within this Peninsular Malaysia population [90]. Moreover, whole genome sequencing has shown evidence of introgression and a mosaic structure of the *P. knowlesi* genome, indicating a breakdown of genetic isolation between divergent groups [85,86,87,88]. This complex population structure should be studied further as it may provide important insights into transmission and pathogenesis in this zoonotic parasite.

In addition to population structure, another area of major interest in *Plasmodium* genome biology is the identification of genes evolving under selective pressures favouring novel alleles or maintaining diversity within populations. The effects and, importantly, the targets of specific forces like anti-malarial drug exposure or protective adaptive immunity can be identified. Prior to large-scale genomic re-sequencing, considerable efforts were expended in disentangling the effects of selection and population processes (such as population subdivision or population expansion) on population genetic indices, such as Tajima’s D, based on population allele frequencies (for a review see [92]). Genome-scale data allows population effects (that will tend to affect the whole genome) and selection effects (the effects of which are more localised) to be separated more easily, as gene or genome region indices can be compared to the distribution of the index across the genome in order to identify outliers. Such whole genome scans for selective sweeps—genome regions with reduced levels of genetic diversity or heterozygosity, or elevated LD—pinpoint chromosomal regions associated with drug-resistance, clearly visible in continent-wide patterns of *P. falciparum* genomic diversity [57]. These characteristic signatures mark loci associated with resistance to different antimalarial drugs. The *dhfr* and *dhps* genes, encoding targets of sulfadoxine and pyrimethamine (used in combination as sulfadoxine-pyrimethamine; SP) show clear signatures of recent strong selection in both *P. falciparum* [93,94] and *P. vivax* [95,96,97], even where SP is not a first line treatment of vivax malaria, possibly showing an ‘off-target’ effect of falciparum malaria treatment. The chloroquine resistance gene *crt* shows similar signatures of selection on resistance alleles in *P. falciparum* [93], while gene amplification has been reported at the *crt* and multidrug resistance gene *mdr1* loci [96,98,99]. In addition to this, recent selection on the region upstream of *crt* has also been reported in Ethiopian *P. vivax* [100].

In the case of resistance to the artemisinin, antimalarials the picture is complex. The first descriptions of slower *P. falciparum* parasite clearance in response to artemisinin were from Cambodia, and subsequently the phenotype spread across southeast Asia [101,102,103]. Numerous studies have associated resistance with mutations in the *kelch13* gene on *P. falciparum* chromosome 13 [101,104,105,106,107], though a large number of different *kelch13* mutations exist [108] and cases of slow parasite clearance have been described in Africa in the absence of known *kelch13* resistance makers [109]. The precise role of *kelch13* in resistance is unclear and is the subject of intense research. Indeed, the mechanism of resistance to artemisinins may differ from that to other drugs in being broader and involving changes to many cellular processes affecting the cell cycle and cellular housekeeping and stress-response processes including the unfolded protein response [110,111,112,113]. Such a broad response can complicate the search for reliable genetic markers of resistance, markers that are vitally important for monitoring the spread of the phenotype from southeast Asia to sub-Saharan Africa. Alongside *kelch13* variants, large scale genomic re-sequencing of southeast Asian parasite populations associated the resistance phenotype with variants in several other genes (*fd*, *mdr2*, *crt*, *arps10*) [105]. Another large-scale re-sequencing study showed 155 SNPs within *kelch13* of which 128 were found in African and 62 in southeast Asian parasites [47]. Analysis of their genetic backgrounds, a key benefit of the whole genome sequencing approach, indicated that the African variants arose independently within Africa, rather than being transferred from southeast Asia, yet they do not appear to be associated with artemisinin resistance in Africa [47]. It has been suggested that certain genetic backgrounds, common in southeast Asia but not Africa, may predispose for *kelch13* resistance mutations and explain the association seen with variants in several other genes [105].

Genome-scale scans of indices of divergence among populations can pinpoint genes under differential selection among these populations. For example, *P. falciparum* populations from countries with differing transmission patterns show unusually high divergence (as measured by the fixation index *F_ST_*) coinciding with a gene (*gdv1*) associated with sexual differentiation [114,115]. In addition to this, apparent differential selection in *crt* was seen between two west African countries: Gambia and Guinea [114,115]. Selection driven by other pressures can also be detected in the genomes of parasites. For example, the globalisation of *P. falciparum* required adaptation to different vector species. Consistent with this, genes expressed in the mosquito stages of the parasite’s life cycle are among the most geographically divergent in the genome [48] and signatures of adaptation are visible in a number of these genes [116]. 

## 4. The Dynamic Genome: Methylomics in *Plasmodium*

Genomics has allowed us to understand *Plasmodium* population level processes in unprecedented detail. However, other ‘omes’ also offer useful windows into aspects of the parasite’s biology that cannot always be studied using genetic variation alone. Understanding how malaria parasites regulate their gene expression is crucial to comprehend *Plasmodium* biology, from the radical changes between life-cycle stages to the expression of alternative invasion pathway proteins and other host-parasite interaction proteins. One way to study this is by transcriptomic quantification of expressed RNA [117,118,119,120,121,122]; another is by epigenomics: mapping of epigenetic marks in the DNA or chromatin structure of the genome [123,124,125,126,127,128]. DNA methylation in *Plasmodium* represents a potential new frontier in *Plasmodium* genomics, yet it remains far from being fully understood and its existence has been somewhat controversial. Here, we consider DNA methylation in *P. falciparum*.

During erythrocyte infection the *P. falciparum* genome is mostly euchromatic [129,130]. Nevertheless, epigenetic mechanisms to control gene expression involving non-coding RNAs, the use of alternative histone variants and histone post-translational modifications are well documented [123,124,125]. Whether the *Plasmodium* epigenetic machinery also included DNA methylation, and in particular 5-methylcytosine (5mC), was a question that awaited an answer for a long time. The apparent lack of 5mC added to other characteristics of *Plasmodium*: lack of a functional RNA interference system [131] and linker histone H1 [131,132] and a mainly euchromatic genome [129,130]. Moreover, *Plasmodium* would not be the only eukaryote dispensing with 5mC: the fruit fly *Drosophila melanogaster* presents an extremely low level (less than 0.01%) of 5mC [133] and *Caenorhabditis elegans*, *Trichoplax adhaerens*, *Schmidtea mediterranea*, the urochordate *Oikopleura dioica*, the rotifer *Adineta vaga* have all dispensed with 5mC [134,135].

However, in eukaryotes 5mC plays a major role in many different processes including genomic imprinting, DNA repair, response to stress and the environment, regulation of gene expression and immune response [133,136,137,138,139]. The AT-rich *Plasmodium* genome [1] represents an obstacle for mass spectrometry-based methods to detect 5mC [140,141] and early analyses did not detect it [140]. Even though approaches that utilize methylation sensitive restriction enzymes (cutting at CG rich sites) and immunoprecipitation are not ideal to properly interrogate the AT-rich *Plasmodium* genome, a methylation-sensitive restriction approach identified a signature of 5mC in the *Dihydrofolate Reductase Thymidylate Synthase* (*DHFR-TS*) gene [142].

Recent advances in the sensitivity of mass spectrometry and the sequencing of bisulfite treated DNA have allowed better investigation of 5mC in *Plasmodium* [126,127,128]. Bisulfite treatment converts unmethylated cytosines into uracils and permits differentiation between methylated and unmethylated sites [143]. It is possible, therefore, sequencing the bisulfite converted DNA, to discern between methylated-cytosines (read as C) and unmethylated-cytosine (read as T). Comparing the sequence of the bisulfite treated genome with a reference genome it is possible to identify methylated and unmethylated cytosines and the proportion of cytosines in the genome that are methylated. In addition, at every cytosine site the proportions of reads containing methylated and unmethylated cytosine can be seen, indicating the proportion of cells in a sample that are methylated at a given position. Using liquid chromatography-tandem mass spectrometry (LC-MS/MS), Ponts and collaborators were able to clearly identify signatures of 5mC in the *P. falciparum* genome and suggested between 0.36% and 1.31% of all cytosines were methylated, depending on the stage analysed (ring, trophozoite or schizont) [128]. This was corroborated by deep sequencing of bisulfite treated *P. falciparum* DNA recovered from an asynchronous population that returned 0.58% methylated cytosines [128]. McInroy and colleagues found a remarkably similar pattern of DNA methylation in the rodent parasite *Plasmodium berghei* [127]. They used LC-MS/MS and a PCR-free bisulfite sequencing method (‘recovery after bisulfite treatment’; ReBuilT) to investigate the *P. berghei* genome for signatures of 5mC. Mass spectrometry and ReBuilT indicated, respectively, 0.33% and 1.87% of all cytosines were methylated. 

**Figure 3:**
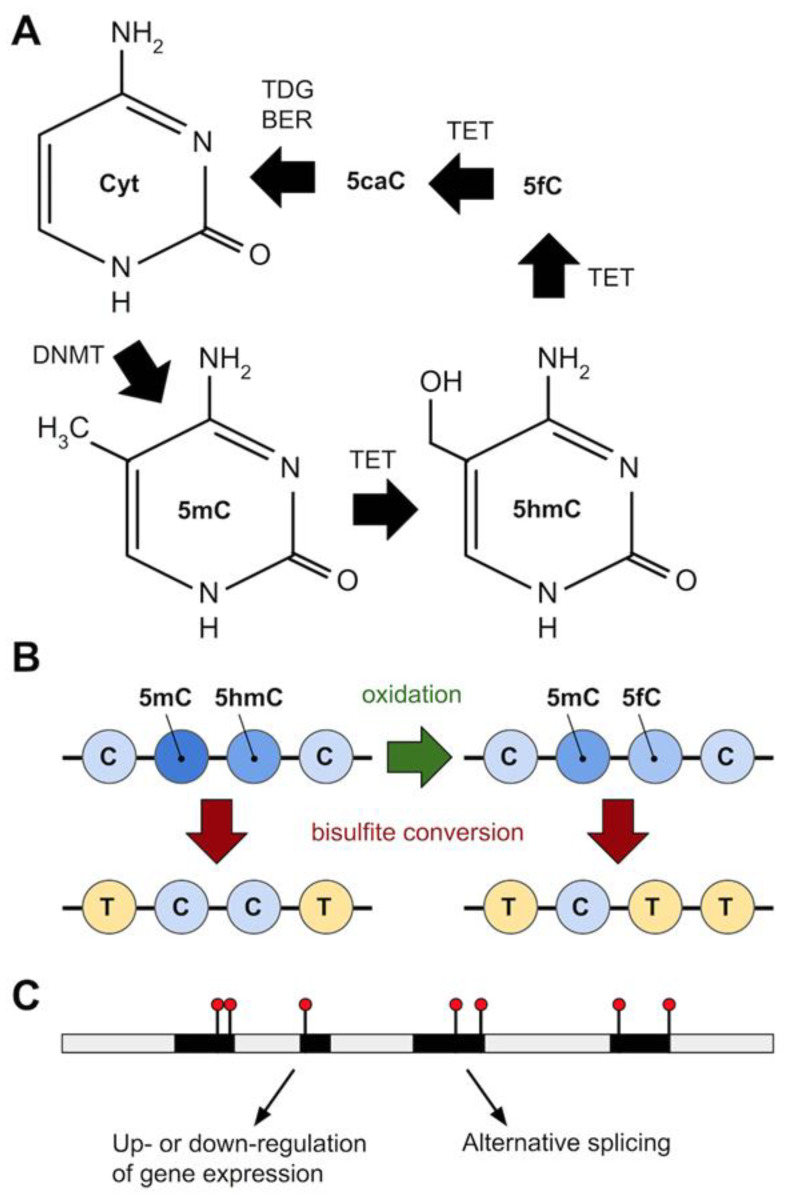
Epigenomics in *Plasmodium*. (**A**) Cytosine can be converted to 5-Methylcytosine (5mC) by DNA methyltransferase (DNMT), and 5mC to 5-Hydroxymethylcytosine (5hmC) by Ten-eleven translocation methylcytosine dioxygenase (TET), which also converts 5hmC to 5-formylcytosine (5fC) and 5fC to 5-carboxylcytosine (5caC). Uracil-DNA glycosylase (TDG) and base-excision repair (BER) converts 5caC back to (unmethylated) cytosine. (**B**) The standard genomic method to detect 5mC (bisulfite treatment to convert unmethylated C to uracil, seen as a T in the final sequencing library, followed by whole genome sequencing) cannot distinguish 5mC from 5hmC. The addition of an oxidation step, converting 5hmC to 5fC (which bisulfite treatment *does* convert to uracil) can allow 5hmC to be detected and quantified. This approach suggests the majority of methylated C in *Plasmodium* is 5hmC. (**C**) Epigenetic marks (black lines with red circles) are shown on a stretch of DNA (grey = intergenic/intronic; black = exonic). In *Plasmodium*, exonic sequence near to intron-exon boundaries is enriched for these modified cytosines. Possible functional consequences include gene silencing or upregulation, and alternative splicing. Genome-wide patterns of methylation could act as alternative markers for gene expression.

Recent findings suggest that the majority of modified cytosines in *P. falciparum* may not in fact be 5mC, but 5-hydroxymethylcytosine (5hmC) [126]. This modified cytosine is derived from the Ten-Eleven Translocation methylcytosine dioxygenase (TET) mediated oxidation of the methyl group (-CH_3_ -> CH_2_OH) of 5mC [144] (Figure 3). TET is part of the pathway to restore unmethylated cytosine: TET catalyzes the conversion of 5mC to 5-carboxylcytosine (5caC), via the intermediates 5hmC and 5-formylcytosine (5fC) [145,146,147,148]; then, uracil-DNA glycosylase and base-excision repair (BER) excises and repairs 5caC back to unmethylated cytosine [145,146,147,149] (Figure 3A). Hammam and colleagues used a variety of methods to query methylation levels in *P. falciparum*, including ELISA, bisulfite sequencing (BS-seq) and oxidative bisulfite sequencing (oxBS-seq), hydroxymethylated DNA immunoprecipitation (hmeDIP) and LC-MS mass spectrometry [126]. Using anti-5mC or anti-5hmC antibodies in different *Plasmodium* life cycle stages (Ring, Trophozoite, Schizont) and ELISA, they estimated 0.19–0.38% cytosine to be 5hmC and only 0.01–0.02% to be 5mC [126]. These proportions were corroborated using a combination of BS-seq and oxBS-seq (0.2–0.26% 5hmC-like and 0.01–0.02% 5mC) [126]. The bisulfite reaction that is part of the BS-seq protocol is unable to convert 5mC or 5hmC into uracil, therefore BS-seq cannot distinguish between these 2 cytosine modifications [150,151]. However, in oxBS-seq 5hmC is first oxidised to 5fC that bisulfite treatment can convert to uracil allowing, by comparing BS-seq and oxBS-seq results, the level of 5hmC to be quantified [150,151] (Figure 3B). Hydroxymethylated DNA immunoprecipitation (hmeDIP) followed by next generation sequencing [152,153] showed the vast majority (98–98.5%) of 5hmC occurred in genic regions while only around 1–1.5% was intergenic, and that most was found in a CHH context (H representing any nucleotide but G) [126]. Further, hydroxymethylated genes had high levels of mRNA expression in all life-cycle stages analysed in the study [126]. Oddly, LC-MS did not identify 5hmC but gave a similar signal that could belong to a more hydrophobic positional isomer (5hmC-like modification) [126]. Overall, Hammam et al.’s [126] results corroborated previous findings on the level and distribution of DNA methylation in *Plasmodium* [127,128], but suggest also that the predominant cytosine modification is 5hmC (or 5hmC-like).

The mechanistic role of 5mC in *Plasmodium* is not clear (Figure 3C). In mammals, most 5mC are found in CG duplexes; however, that is not the case in *P. falciparum* where the vast majority of 5mC are found in CHH contexts [128]. This is also seen in *P. berghei* (CAH is particularly common), where only a small proportion was associated with CG, CC or CHG [127]. The distribution of methylation in *P. falciparum*, with more methylation in the gene body than in intergenic regions and demarcating exon-intron boundaries, is also seen in other lineages [135] and may suggest a role in regulating alternative splicing [154] or RNA polymerase II DNA binding affinity [155]. In *P. berghei*, methylation seems to be equally distributed between genic and intergenic regions though within genes methylation seems to be mostly exonic and to mark intron–exon boundaries [127].

There seems to be a correlation between the distribution of 5mC and specific histone modifications in *P. falciparum*. Heterochromatin is characterized by H3K9me3 while transcriptionally active euchromatin is marked by H3K9ac and H4K20me3, at least in *var* genes [123,124,125]. H3K9ac and H3K4me3 associate to the coding region of actively expressed genes while H3K36me2 is a marker of repression. Cytosine methylation levels increase downstream of histone markers (H3K9ac and H3K4me3) linked to higher expression, while methylation levels do not change around silencing histone markers (H3K9me3 and H4K20me3) [128]. It is important to note here, that in many metazoans, H3K4me3 anticorrelates with 5mC due to the reduced affinity of DNMT3 ADD domain for this histone modification [156,157].

The fact that methylation profiles are different between DNA strands and that methylation levels change among life cycle stages suggest most if not all 5mC in *Plasmodium* is *de novo* methylation [128]. This implies that *Plasmodium* must express enzymes to add and remove 5mC. The eukaryotic methylation toolkit includes six DNA Methyltransferases (DNMTs) with different domains and different phylogenetic relationships, three Ten-Eleven Translocation methylcytosine dioxygenase (TET) and eleven Methyl-CpG Binding Domain (MBD) readers [135,145,147]. Some eukaryotic lineages have duplicated or lost some of the main DNMTs and other components of the methylation toolkit [135]. Initial investigations identified only one DNMT2 in the *Plasmodium* genome [128]. In mammals and other eukaryotes, different DNMTs have dedicated roles. DNMT1 seems to be the dedicated ‘maintenance methyltransferase’, replicating the methylation signature to newly formed DNA strands after cell division [135,145,147]. DNMT3 is the dedicated ‘*de novo* methyltransferase’, adding new methyl markers, often asymmetrically, to DNA strands. DNMT2 is evolutionarily highly conserved, but its role seems to be associated to tRNA methylation and species that only express this methylases often lack 5mC [145,158]. Indeed, *Plasmodium* aspartic tRNA is methylated by *Plasmodium* DNMT2 (TRDMT1) [159]. Importantly, Hammam and colleagues, using ELISA, demonstrated TET hydroxylase activity in *Plasmodium* nuclear extracts however they could not identify a TET homologous in the *P. falciparum* genome [126].

*Plasmodium* methylation context (mostly CHH) may suggest possible components of its methylation system. Similarities and differences between eukaryotic lineages suggest a great diversity and plasticity in the evolution of mechanisms of 5mC and its function [135]. For example, animals have well conserved DNMT1 and DNMT3 genes but display substantial differences in the distribution and level of 5mC, ranging from hypermethylated to sparsely methylated genomes in mammals and hymenoptera respectively [135]. DNMT5 mediates nucleosomal linkers methylation in some marine algae, but this pattern is not replicated in DNMT5-expressing fungi [160,161] and in some plants DNMT3 only methylates CHH context [162]. This suggests that the particular pattern of methylation observed in an organism may be the results of the activity of modulators (e.g., MDB proteins) that control DNMT activity. It is also possible that methylated cytosines in CHH context serve as binding sites for MDB proteins [147]. In mammal brains MeCP2 (an MDB protein family member) binds 5mC in non-CpG context and regulates gene expression [163,164,165,166].

The recent discovery that the more abundant cytosine modification in *P. falciparum* is indeed 5hmC may have implications for how this epigenetic marker modulates gene expression. The 5hmC signature is read by specific members of the MBD protein family such as methyl-CpG binding protein2 (Mecp2), methyl-CpG binding domain proteins 4 (Mbd4), Kaiso and SRA (reviewed in [167]). Mbd4 was also implicated in the process of demethylation converting 5hmC in 5hmUracil by deamination [168]. The 5hmC was also found to be particularly enriched in embryonic stem cells and brain tissues in euchromatic regions and in gene bodies, where its level correlated with gene expression [153,168,169,170,171,172].

It is becoming apparent that *Plasmodium* epigenetic machinery does include DNA methylation but much remains unknown. Bioinformatic approaches have at times been insufficient to identify *Plasmodium* orthologs [1] and these approaches failed to clearly identify the components of *Plasmodium* methylation/demethylation pathway. It is possible that MDB proteins play fundamental roles in modulating DNA methylation/demethylation in *Plasmodium* and that may even be the crucial component of this pathway. The role of 5hmC, as well as 5fC, needs further consideration. 5fC was found at promoters of active genes and in association with H3K4me3 in ES cells, again underlining the connection between cytosine modifications, histone modifications and gene expression [173,174,175]. Methodological advances are helping to overcome some of the limitations imposed by *Plasmodium*’s AT-rich genome on fully investigating the role of cytosine modification. For example, the PCR-free ‘ReBuilT’ approach used by McInroy et al. [127] recovers many sequence fragments that are usually lost during bisulfite sequencing library preparation and avoids PCR amplification biases (favouring GC-rich DNA).

## 5. Conclusions

Recent years have seen huge advances in *Plasmodium* genomics. Through intensive and extensive study of the genomes of these parasites, we have learned a great deal about their origins, evolution and biology. Future prospects include the further embedding of genomics in malaria epidemiology as well as advances in, and possible large-scale analysis of, the other ‘omics of malaria to gain further insights into this deadly, fascinating parasite.

## Data Availability

No new data were created or analyzed in this study.
